# Cardiometabolic effects of hypoprolactinemia

**DOI:** 10.1007/s11154-024-09891-z

**Published:** 2024-07-30

**Authors:** Renata S. Auriemma, Roberta Scairati, Rosa Pirchio, Guendalina Del Vecchio, Sara Di Meglio, Davide Menafra, Rosario Pivonello, Annamaria Colao

**Affiliations:** 1 Dipartimento di Medicina Clinica e Chirurgia, Sezione di Endocrinologia, Diabetologia, Andrologia e Nutrizione, Università Federico II di Napoli, Via Sergio Pansini 5, 80131 Naples, Italy; 2 Dipartimento di Medicina Clinica e Chirurgia, Sezione di Endocrinologia, Diabetologia, Andrologia e Nutrizione, Unità di Andrologia e Medicina della Riproduzione, Sessualità e Affermazione di Genere, Università Federico II di Napoli, Naples, Italy; 3 Unesco Chair for Health Education and Sustainable Development, “Federico II” University, Naples, Italy

**Keywords:** Hypoprolactinemia, Dopamine agonists, Cabergoline, Insulin resistance, Metabolic syndrome, Cardiovascular disease

## Abstract

The fall of PRL levels below the lower limit of the normal range configures the condition of hypoprolactinemia. Unlike PRL excess, whose clinical features and treatments are well established, hypoprolactinemia has been only recently described as a morbid entity requiring prompt identification and proper therapeutic approach. Particularly, hypoprolactinemia has been reported to be associated with the development of metabolic syndrome and impaired cardiometabolic health, as visceral obesity, insulin-resistance, diabetes mellitus, dyslipidaemia, chronic inflammation, and sexual dysfunction have been found more prevalent in patients with hypoprolactinemia as compared to those with normoprolactinemia. This evidence has been collected mainly in patients on chronic treatment with dopamine agonists for PRL excess due to a PRL-secreting pituitary tumour, and less frequently in those receiving the atypical antipsychotic aripiprazole. Nowadays, hypoprolactinemia appears to represent a novel and unexpected risk factor for cardiovascular diseases, as is the case for hyperprolactinemia. Nevertheless, current knowledge still lacks an accurate biochemical definition of hypoprolactinemia, since no clear PRL threshold has been established to rule in the diagnosis of PRL deficiency enabling early identification of those individual subjects with increased cardiovascular risk directly ascribable to the hormonal imbalance. The current review article focuses on the effects of hypoprolactinemia on the modulation of body weight, gluco-insulinemic and lipid profile, and provides latest knowledge about potential cardiovascular outcomes of hypoprolactinemia.

## Introduction

Cardiometabolic homeostasis is strongly influenced by hormonal balance, and in turn hormonal status highly prejudices cardiometabolic well-being. In fact, many metabolic diseases, mainly including obesity, diabetes mellitus, dyslipidemia, non-alcoholic fatty liver disease (NAFLD), and metabolic syndrome, are known to severely affect cardiometabolic health and to promote major adverse cardiovascular events [1]. Over the last thirty years, increasing body of research has documented that prolactin (PRL) plays a key role in the modulation of metabolic homeostasis, emphasizing in both animal and human models that abnormal PRL secretion is metabolically detrimental [2, 3]. The evidence of PRL receptor expression on either β-cells in pancreatic islets and adipocytes in adipose tissue provided the basis to clarify the effects of PRL on global metabolic balance. PRL receptors expression has been demonstrated on insulin-secreting cell lines [4] as well as in rat [5] and human [6] pancreatic β-cells, and PRL reportedly increases β-cell proliferation, anti-apoptotic activity, insulin gene transcription, and glucose-dependent insulin secretion in rats and humans [7–11]. The enhanced expression of PRL receptors on pancreatic β-cells demonstrated during pregnancy [12] has been reported to be essential to enable PRL direct actions on metabolic changes of pregnancy, including hyperphagia, increased adiposity, physiological insulin resistance, and ultimately on carbohydrate homeostasis, to ensure a proper source of glucose to the fetal-placental unit [7].

To a similar extent, PRL receptors have been found to increase during adipocyte differentiation and may be involved in lipid metabolism of mature adipocytes [13, 14]. Indeed, in mouse cell lines PRL has been shown to play a crucial role in the adipogenic stem cell differentiation and has been suggested to be involved in the *beigeing* of white adipose tissue [15, 16], whereas in human adipocytes PRL has been found to inhibit lipogenesis [17]. Additionally, PRL has been demonstrated to inhibit adiponectin serum concentration in both humans and mice [18], and to increase leptin release in rat and mouse models [19, 20], thus leading to leptin resistance.

Altogether, this evidence has suggested that PRL exerts a crucial role in the control of gluco-insulinemic and lipid metabolism, raising the question of whether abnormally increased or reduced PRL levels might impair metabolic health.

Data collected in patients with PRL excess due to a PRL-secreting pituitary tumour have evidently demonstrated that hyperprolactinemia is associated with an impaired gluco-insulinemic and lipid profile [21–38], accountable for the development of overt metabolic syndrome in approximately half of patients [33], irrespectively of concomitant hypogonadism [39], thus supporting the hypothesis that PRL excess may trigger an increased cardiovascular risk [40, 41]. Particularly, PRL values above 100 ng/ml have been proposed to be associated with an increased prevalence of obesity, glucose intolerance, insulin resistance and metabolic syndrome [42].

Interestingly, more recently similar negative metabolic outcomes have been described also in patients with very low PRL levels (i.e., below 7 ng/ml), in whom PRL deficiency has been hypothesized to be associated with an increased prevalence of type 2 diabetes mellitus [43–45], metabolic syndrome [46, 47], insulin resistance [45], NAFLD [48] and obesity [49]. In this light, a new pathologic entity, namely *hypoprolactinemia*, may be considered an adjunctive risk factor for cardiovascular disease.

Based on these findings, a metabolically healthy window of PRL levels ranging 25–100 ng/ml, also known as *homeostatic functionally increased transient prolactinemia* (HomeoFIT-PRL), has been proposed as a functional response necessary for maintaining metabolic homeostasis in challenging physiological situations, such as stress, hypoglycaemia, and exercise, even in absence of pathologic conditions [42].

However, despite the growing interest in the long-term cardiovascular consequences of hyperprolactinemia, cardiometabolic impact of hypoprolactinemia is less known and not extensively investigated, and studies specifically focusing on the link between hypoprolactinemia and cardiovascular health are still too scant.

The current review article focuses on the effects of hypoprolactinemia on the modulation of body weight, gluco-insulinemic and lipid profile, and provides latest knowledge about potential cardiovascular outcomes of hypoprolactinemia.

## Hypoprolactinemia, body weight, glucose homeostasis and lipid profile: evidence from animal models

Long-term consequences of hypoprolactinemia on body composition and gluco-insulinemic and lipid profile have been investigated in animal models to elucidate molecular mechanisms responsible for potential implications of PRL deficiency on the development of metabolic syndrome and impaired cardiovascular health [50, 51, 49, 52].

In non-obese diabetic female mice, the daily administration of bromocriptine 200 μg from 21 days of age (i.e., the time of weaning) to 112 days of age, leading to the condition of hypoprolactinemia, has been found to result in a significantly lower incidence (40.9%) of type 1 diabetes mellitus as compared to control animals (81.5%) at 112 days [50]. Interestingly, bromocriptine injections did not produce a comparable effect in male mice, in whom the occurrence of type 1 diabetes mellitus was found to increase in the opposite conditions of hyperprolactinemia induced by syngeneic anterior pituitary transplant [50]. Following treatment with bromocriptine, PRL levels were < 5 ng/ml at either 28, 56 and 112 days of treatment in male mice, and between 10 and 15 ng/ml at the same time-points in female mice, thus suggesting that hypoprolactinemia severity might variably impact the incidence of autoimmune diseases, such as type 1 diabetes mellitus, in both sexes [50].

In another study on lactating rats, bromocriptine-induced maternal hypoprolactinemia induced unfavourable metabolic effects by affecting metabolic programming for adult offspring [51, 52]. In fact, the suppression of milk production induced by bromocriptine has been shown to cause neonatal offspring malnutrition and to program for obesity, hyperphagia, and hyperleptinemia with leptin resistance in adult offspring [51]. In lactating rats treated with bromocriptine 1 mg twice a day, or saline (controls), for the last 3 days of lactation, body weight and food intake were monitored up to the day of sacrifice at 180 days [52]. Compared to controls, adult offspring whose mothers were treated with bromocriptine had higher body weight and total, visceral and subcutaneous body fat, associated with hyperglycemia, lower muscle glycogen, hypercholesterolemia due to a significant increase in LDL-cholesterol and decrease in HDL-cholesterol, hypertriglyceridemia, leptin resistance coupled with lower serum adiponectin concentrations, and insulin resistance [52]. Interestingly, the injection of leptin in bromocriptine-treated rats did not affect food intake at 2, 4 and 6 h, thus suggesting that the increase in body weight was not consequent to the increase of food ingestion, but to a hypometabolic state [52].

Altogether, these findings suggested that inadequate PRL suppression by dopamine agonists might alter metabolic homeostasis, triggering excessive body fat accumulation, insulin resistance and metabolic syndrome, and thus promoting the increase in overall cardiovascular risk.

This assumption is reinforced by the evidence that PRL receptor-null mice fed with high fat diet (HFD) gained more body weight and acquired a greater fat mass due to a marked adipocyte hypertrophy not associated with significant changes in adipocyte number, also developing insulin resistance and glucose intolerance, as compared with wild-type mice [49]. Following treatment with PRL, HFD-fed mice displayed a significant improvement in insulin-induced glucose-lowering effect during an insulin tolerance test and a significant reduction in adipocyte hypertrophy in visceral adipose tissue as compared with HFD-fed animals not receiving treatment with PRL and control diet-fed animals [49], therefore leading to the conclusion that in obesity conditions PRL plays a crucial role in the promotion of a healthier form of adipose tissue growth with enhanced metabolic performance [49]. Indeed, in visceral, but not subcutaneous, adipose tissue the expression of peroxisome proliferator-activated receptor gamma (PPAR-γ) was found to significantly increase, and that of interleukin (IL)-1b to significantly reduce after treatment with PRL [49], thereby suggesting that the correction of hypoprolactinemia might improve peripheral insulin sensitivity, fatty acid storage and glucose metabolism, and limit the impact of chronic inflammation characteristic of fat tissue [49], which reportedly contributes to the development of insulin resistance [53].

**Fig. 1:**
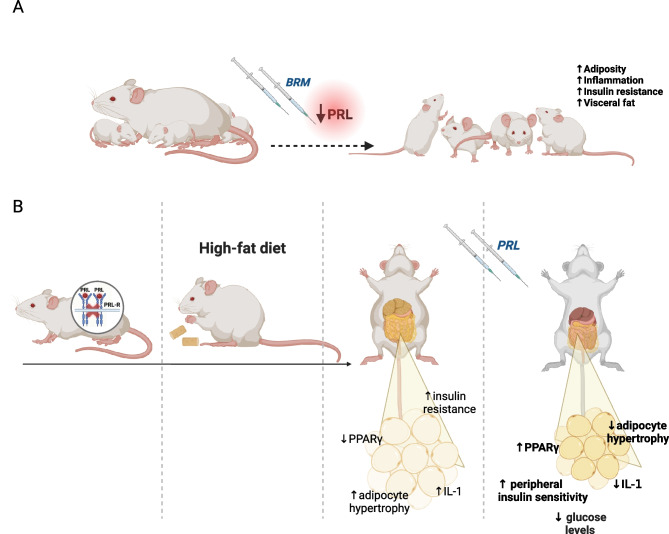
Results from studies in animal models investigating the effects of hypoprolactinemia on body weight, glucose homeostasis and lipid profile. Panel **A**: the administration of bromocriptine (BRM) to lactating rats, resulting in the suppression of milk production, promoted obesity, hyperphagia, and hyperleptinemia with leptin resistance in adult offspring leading to weight gain, increased adiposity, mainly at visceral level, and insulin resistance. Panel **B**: PRL receptor-null mice fed with high fat diet gained more body weight, acquired a greater fat mass due to a marked adipocyte hypertrophy and developed insulin resistance and glucose intolerance, as compared with wild-type mice. PRL administration resulted in the significant reduction in adipocyte hypertrophy in visceral adipose tissue, associated with higher expression of PPAR-γ, reduced release of IL-1 and decreased plasma glucose levels, thus improving peripheral insulin sensitivity. Abbreviations: PPAR-γ, peroxisome proliferator-activated receptor gamma; IL-1, interleukin-1. Created with Biorender.com

Cardiometabolic effects of hypoprolactinemia in animal models are summarized in Fig. 1.

## Pathophysiology and biochemical definition of hypoprolactinemia

PRL deficiency, or hypoprolactinemia, consists of very low or undetectable PRL levels deriving from a pituitary dysfunction caused by genetic causes, or acquired from pituitary tumors, neurosurgery, pituitary irradiation, chemotherapy, infiltrative disorders, autoimmune diseases, traumatic brain injuries, vascular damage, and drugs, as is the case of any isolated pituitary deficiency [54].

Idiopathic isolated hypoprolactinemia is exceptionally rare and hardly described in the literature [55–60]. Albeit rare cases of a familial hereditary disease [57] resulting in puerperal alactogenesis with a potential autosomal recessive inheritance [59] have been described, no specific gene mutation responsible for PRL deficiency has been reported to date [54].

The most common origin of hypoprolactinemia is iatrogenic, consequent to medical therapy with the atypical antipsychotic aripiprazole or mostly with dopamine agonists, particularly cabergoline, which represents the cornerstone of treatment of hyperprolactinemia due to PRL-secreting pituitary tumours [61].

The literature on antipsychotic-induced hypoprolactinemia is relatively scarce. The effects of antipsychotic drugs on PRL levels have been recently evaluated in a random effect meta-analysis [62], reporting that hypoprolactinemia occurred in approximately 31% (range 9.5–45.5%) of patients receiving treatment with aripiprazole and less frequently in those treated with asenapine (5.3%, range 3.5–6.7%). Additionally, aripiprazole is known to exert a direct impact on human adipocyte differentiation, as the use of this compound at therapeutic concentrations has been shown to increase the expression of gene markers of fatty acid oxidation [63], thus suggesting that aripiprazole, but not other antipsychotic drugs, might directly alter adipocyte differentiation, potentially triggering a switch from glucose to lipid utilization in human adipocytes [63].

However, available data are limited to few studies, thus implying that current evidence about the association between the use of aripiprazole and the occurrence of hypoprolactinemia is not strong enough to draw definitive conclusions.

Conversely, available evidence in literature about the impact of dopamine agonists on excessive PRL lowering is robust, and several studies have described the occurrence of PRL deficiency following treatment with these compounds. For long time, routine clinical practice for the management of patients with PRL excess has been based on the assumption that low or undetectable PRL levels during treatment with dopamine agonists might be associated with a better prognosis and long-term rescue from the pituitary tumours. Nowadays, PRL levels ≤ 162 mU/L (i.e., 7.6 ng/ml) under low maintenance doses of cabergoline are still considered the biochemical goal to achieve, together with a negative pituitary imaging, before attempting cabergoline withdrawal in patients with prolactinomas [64], thus suggesting that low or undetectable PRL value might favourably predict definitive remission. In this light, the achievement of a > 90% suppression of PRL levels and a substantial tumour shrinkage have been proposed as the main criteria for the systematic evaluation of the outcomes of medical treatment with cabergoline before a trial of drug discontinuation was experimented in clinical settings [65].

More recently, patients reaching low or suppressed PRL levels while on treatment with dopamine agonists have been reported to experience a wide range of adverse clinical implications, including impaired sexual function, delayed ovulation and oligomenorrhea, and increased cardiovascular risk [47, 66–75]. However, available studies investigating clinical consequences of hypoprolactinemia have failed to provide a unique biochemical threshold below which PRL should be considered too low and a diagnosis of PRL deficiency could be ruled in. In fact, several biochemical definitions of hypoprolactinemia have been proposed across studies [47, 66–75], as shown in Table 1. Hence, PRL deficiency has been variably reported to occur when PRL levels fell below 1.8 ng/ml [66], 3 ng/ml [68, 70, 72], 5 ng/ml [71, 73, 74] or even 12 ng/ml [69]. Some studies [47] did not identify a specific cut-off value of PRL to define hypoprolactinemia but suggested the interval quartile ranges of low or undetectable PRL levels which more frequently were associated with adverse clinical outcomes. Particularly, in men with sexual dysfunction PRL levels in the first or the second quartile, corresponding to PRL < 5.0 ng/ml or ranging 5.1–7.0 ng/ml, respectively, were associated with higher rates of metabolic syndrome, diabetes mellitus, arteriogenic erectile dysfunction, and premature ejaculation [47]. Interestingly, in pre- and post-menopausal women seeking medical advice for sexual dysfunction the lowest PRL quartile ranged 5.12–6.53 ng/ml [75], corresponding to the second PRL quartile in men with sexual dysfunction [47], thus suggesting a potential gender difference in the biochemical definition of hypoprolactinemia, as already recognized for normoprolactinemia [61].

**Table 1:** Overview of biochemical definitions and clinical implications of hypoprolactinemia across studies

**Reference nr**	**Author, yr**	**Subject nr**	**Subject characteristics**	**PRL levels (ng/ml)**	**Clinical implications of PRL deficiency**
[66]	Mukherjee, 2006	162	Hypopituitarism	< 1.8	Predictor of GH deficiency severity in patients with hypopituitarism
[47]	Corona, 2009	2496	Men with sexual dysfunction	< 5.0 (I quartile) 5.1–7 (II quartile)	↑metabolic syndrome rate ↑arteriogenic erectile dysfunction rate ↑premature ejaculation rate ↑diabetes mellitus rate
[67]	Maseroli, 2015	3847 (UNIFI) 202 (EMAS)	Erectile dysfunction	< 5.0	-
[68]	Sogawa, 2016	25	13 men and 12 women receiving aripiprazole (mean dose 10.8 mg)	< 3.57 in men < 6.12 in women	-
[69]	Ponce, 2020	40	Abdominal surgery	< 12	↑fasting insulin levels ↑HOMA-IR ↓HOMA-S ↑visceral adiposity ↑adipocyte hypertrophy index ↑ adipocyte size
[70]	Krysiak, 2020	50	Premenopausal women on chronic CAB	3.2 ± 1.5	↓ FSFI score ↑ BDI-II score
[71]	Tasaki, 2020	177	Men on aripiprazole	< 5.0	-
[72]	Krysiak, 2021	56	Men on chronic DA	< 3.0	↓ total and free testosterone ↓ erectile function score ↓ sexual desire score ↑ BDI-II score
[73]	Krysiak, 2022	51	26 premenopausal CAB treated patients on chronic atorvastatine *vs* 25 controls	< 5.0	↑ HOMA-IR ↑ total and LDL-cholesterol ↑ uric acid ↑ C-reactive protein ↑ fibrinogen ↑ homocysteine ↓ 25-hydroxyvitamin D
[74]	Krysiak, 2022	67	39 premenopausal CAB treated patients *vs* 28 controls	< 5.0	↑2 h-post load glucose ↑ HbA_1c_ ↑ triglycerides ↑ uric acid ↑ C-reactive protein ↑ fibrinogen ↓ HDL-cholesterol
[75]	Maseroli, 2023	319	277 pre- and post-menopausal women with sexual dysfunction *vs* 42 controls	5.12–6.53 (I quartile)	↑ BMI ↑ waist circumference ↑ systolic blood pressure ↑ FSH and LH ↑ total and LDL-cholesterol ↑ HSDD prevalence

*GH* growth hormone, *UNIFI* University of Florence, *EMAS* European Male Aging Study, *CAB* cabergoline, *HOMA-IR* Homeostasis model assessment of insulin resistance, *HOMA-S* Homeostasis model assessment of insulin sensitivity, *FSFI* female sexual functioning index, *BDI-II* Beck Depression Inventory-II, *DA* dopamine agonists, *BMI* body mass index, *HSDD* hypoactive sexual desire disorder

## Cardiometabolic impact of hypoprolactinemia

**Fig. 2:**
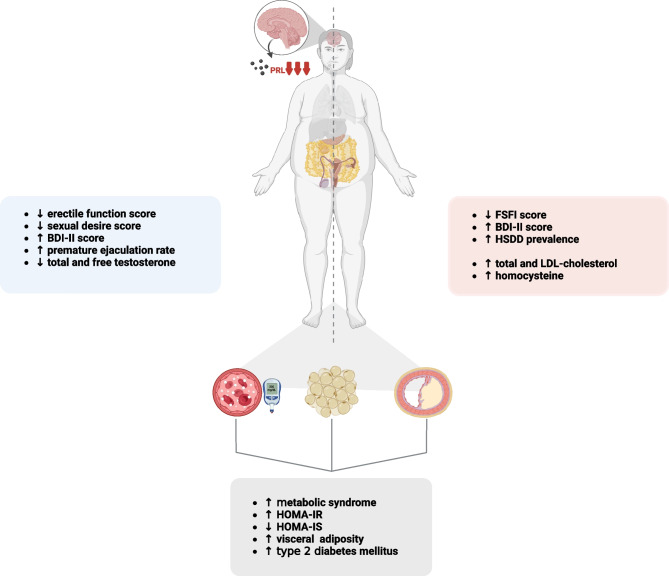
Results from studies in humans investigating the effects of hypoprolactinemia on body weight, glucose homeostasis and lipid profile. In both genders, PRL deficiency alters global metabolic homeostasis by promoting peripheral insulin resistance and visceral adiposity, thus increasing the rate of metabolic syndrome and diabetes mellitus. These metabolic effects result in gender-specific clinical features of hypoprolactinemia, which are strongly related to sexual and global psychological health. In men (left side), PRL deficiency is associated with sexual dysfunction due to erectile dysfunction, low sexual desire, and premature ejaculation, which results further impaired in patients with concomitant hypogonadism. In women (right side) hypoprolactinemia is associated with sexual dysfunction and hypoactive sexual desire disorder. Consequently, both men and women experience raising depressive symptoms and altered body image perception. Created with Biorender.com

Independently on the PRL levels adopted for the biochemical definition of hypoprolactinemia, available studies [47, 66–75] have consistently reported that PRL deficiency negatively affects cardiometabolic health, as shown in Fig. 2. Undeniably, low or suppressed PRL levels have been shown to increase the rate of metabolic syndrome [47, 69, 73–75]. Indeed, most patients with hypoprolactinemia displayed higher BMI and waist circumference [75], fasting insulin levels [69], HOMA-IR [69, 73], HbA_1c_ [74], visceral adiposity [69], lipid fractions [73, 74] and homocysteine [73] as compared to those with PRL levels within the normal range, regardless from the aetiology of hypoprolactinemia (hypopituitarism, or medical treatment with dopamine agonists). Additionally, PRL levels have been demonstrated to inversely correlate with 2 h post-load glucose levels [74], HOMA-IR [73, 74], HbA_1c_ [74, 75], triglycerides [74], total and LDL-cholesterol [75], uric acid [73], C-reactive protein [73, 74], fibrinogen [73, 74] and carotid intima-media thickness [74], and directly with HDL-cholesterol [73, 74] and the quantitative insulin sensitivity check index QUICKI [74]. In brief, PRL deficiency appears to impair peripheral insulin sensitivity and to promote low-grade systemic inflammation and perturbed coagulation, ultimately altering cardiometabolic parameters and exposing the patients to a greater cardiovascular risk. Noteworthy, the negative impact of hypoprolactinemia on cardiometabolic health has been documented both in patients receiving [72–74] or not receiving [47, 75] treatment with cabergoline, therefore leading to the conclusion that PRL levels per se, rather than the use of medical therapy with dopamine agonists, might impair cardiometabolic well-being. As a matter of fact, among patients chronically treated with cabergoline for PRL-secreting tumours, impaired cardiometabolic parameters were found exclusively in patients displaying hypoprolactinemia as compared to those achieving normoprolactinemia or cabergoline-naïve [73, 74], and PRL levels have been demonstrated to be the best predictors of hyperglycaemia [47], hypertriglyceridemia [47] and HOMA-IR [69] together with waist circumference, in patients with hypoprolactinemia.

Based on the current knowledge, some detrimental metabolic effects induced by PRL deficiency appear to parallel those induced by PRL excess. Particularly, in both PRL excess and deficiency the main metabolic features are weight gain and predisposition to obesity and insulin resistance. Nevertheless, molecular mechanisms underpinning the changes in body weight and insulinemic profile differ between hyper- and hypoprolactinemia. PRL excess is reportedly associated with increased food intake mainly ascribable to the increase in orexigenic hormones, such as agouti-related peptide and neuropeptide Y in the hypothalamic arcuate and dorsomedial nuclei, respectively, most likely in response to the functional blocking of the dopaminergic tone [2]. The resulting condition of hyperphagia triggers weight gain and increased BMI, and promotes several metabolic abnormalities including increased fat storage, atherogenic lipid profile (increased LDL-cholesterol and triglycerides, together with reduced HDL-cholesterol and apolipoproteins A-I and A-II), insulin and leptin resistance, and reduced adiponectin, thus leading to metabolic syndrome [2]. In the case of hypoprolactinemia, the inadequately low PRL levels necessary for metabolic homeostasis do not affect food intake but promote adipocyte enlargement and adipose tissue hypertrophy, particularly at visceral level, together with a reduced expression of adipocyte markers such as adiponectin and GLUT-4, thus leading to insulin resistance [69], and foster the increase in fasting glucose and insulin levels, HbA_1c_, LDH-cholesterol and triglycerides, and the decrease in HDL-cholesterol [73, 74], thus facilitating the development of type 2 diabetes mellitus and metabolic syndrome.

In this light, an innovative metabolic classification into three PRL interval ranges (hypoprolactinemia, normoprolactinemia and hyperprolactinemia) has been recently proposed in the context of the HomeoFIT-PRL [42]. Hypoprolactinemia, defined as a PRL level < 7 ng/ml, should be considered metabolically detrimental. Normoprolactinemia, defined as a PRL level ranging 7–25 ng/ml, is further subclassified into two intervals: the PRL interval range 7–15 ng/ml should be considered expression of metabolic maintenance, whereas the PRL interval range 15–25 ng/ml appears to be metabolically beneficial. In absence of physiologic or pathologic conditions altering PRL secretion, hyperprolactinemia with PRL levels mildly or moderately above the normal range (25 -100 ng/ml) has been proposed to represent a physiological response to an increase in metabolic demand, as in case of hypoglycaemia, stress, sexual arousal, and intense physical exercise [42]. As a matter of fact, PRL levels ranging 25–40 ng/ml have been found associated with a lower prevalence of type 2 diabetes mellitus [43], metabolic syndrome [47], NAFLD [48], and major cardiovascular events [76].

However, as long as PRL levels increase above the normal range, patients can experience the reduction in FSH and LH secretion, thus leading to hypogonadropic hypogonadism. In this scenario, PRL and sexual hormones should be carefully balanced to minimize the cardiovascular and metabolic detrimental effects of oestrogen and testosterone deficiency.

Altogether, these findings have suggested that in routine clinical practice of patients with PRL excess requiring a PRL-lowering treatment a critical attention should be paid to the adequate reduction of PRL levels not overstepping in hormonal suppression, and a proper dose adjustment of dopamine agonists should be promptly applied to restore PRL levels within the normal range as soon as a condition of hypoprolactinemia is detected. In those individual patients experiencing PRL deficiency independently of dopamine agonist treatment, or in whom these drugs cannot be withdrawn, or down-titrated, healthy lifestyle and nutritional habits should be strongly recommended, also introducing proper medical treatment with insulin-sensitizers, glucose lowering drugs and/or statins in case of very high cardiovascular risk.

Additionally, PRL deficiency has been reported to be independently associated with significantly lower IGF-I status in individuals with severe GH deficiency [66]. In this respect, patients with GH deficiency should be carefully monitored for concomitant hypoprolactinemia, which in turn might aggravate IGF-I deficiency [66], thus exacerbating the increased cardiovascular risk reported in GH deficient patients due to abnormal body composition, unfavourable lipid profile, increased fibrinogen and C-reactive protein levels, insulin resistance, early atherosclerosis and endothelial dysfunction, and impaired left ventricular performance [77]. In association to other pituitary deficiencies, hypoprolactinemia might contribute to further impair cardiometabolic health, although studies specifically addressing the role of PRL deficiency as a risk factor for cardiovascular mortality in patients with hypopituitarism are scant. However, considering the severe cardiovascular and metabolic impact of hypoprolactinemia, it is reasonable to assume that the association of PRL deficiency with cortisol and/or GH deficiency can only increase the overall cardiometabolic risk. Moreover, standard mortality rates have been reported to vary from 0.98 to 3.36 in men and from 2.11 to 4.54 in women with hypopituitarism, and to recover in men, but not in women, receiving replacement treatment [78], thus suggesting that hypopituitary women are at greater mortality risk than hypopituitary men. Whether PRL deficiency might contribute to this apparent gender difference in mortality rates in patients with hypopituitarism requires further investigation.

In patients at high cardiovascular risk, defined as ≥ 5% risk to develop a cardiovascular event within the next 10 years, caution has been recommended for the use of dopamine agonists in addition to the treatment with statins, as hypoprolactinemia may negatively impact and attenuate cardiometabolic preservative effects of statins [73].

In both men and women with hypoprolactinemia, the association between PRL deficiency and impaired sexual function has emerged [47, 70, 72, 75]. Particularly, men with PRL levels < 5.0 ng/ml or ranging 5.1–7 ng/ml showed a higher prevalence of diabetes mellitus, dyslipidaemia, severe erectile dysfunction and partial erection at the International Index of Erectile Function as compared to those with higher PRL levels [47, 72], together with a higher prevalence of mild depression in men with PRL levels below 3 ng/ml under chronic dopamine agonists treatment [72]. Noteworthy, proper adjustment of dopamine agonists dose leading to the recovery of normoprolactinemia significantly increased the scores of erectile function and sexual desire domains and decreased the prevalence of subjects with total or mild depressive symptoms [72].

To a similar extent, at the Female Sexual Functioning Index (FSFI) in women with hypoprolactinemia scores for desire [70, 75], arousal [70] and satisfaction [75] domains were significantly lower, and prevalence of mild depression significantly higher, as compared to those with normoprolactinemia, and PRL levels < 9.83 ng/ml resulted associated with a greater prevalence of hypoactive sexual desire disorder and body image discomfort [75]. In women under chronic treatment with dopamine agonists, adequate dose reduction significantly increased FSFI desire and arousal domain scores, also reducing the overall prevalence of sexual dysfunction [70].

Altogether, these findings suggested that PRL deficiency might impair sexual function in both men and women, mainly affecting sexual desire and raising the occurrence of depressive symptoms and altered body image perception.

These data have crucial clinical implications, because a strong connection between sexual dysfunction and cardiovascular diseases has been clearly demonstrated [79, 80], and in men erectile dysfunction has been reported to predict cardiovascular events as an independent risk factor [81].

In this light, based on the evidence of a causative role of hypoprolactinemia for sexual dysfunction, a careful evaluation of cardiovascular health becomes strongly advisable in those individual patients with a biochemical confirmation of PRL deficiency, therefore prompting adequate therapeutic measures to limit the overall cardiovascular risk in these subjects. In turn, patients at high cardiovascular risk should be screened for PRL levels, thus allowing the early identification of subjects with PRL unbalance.

## Conclusions

PRL has a major role in the control of global metabolic homeostasis. Sustained PRL levels within the normal range are crucial for cardiometabolic health, as abnormal PRL levels might represent a risk factor for the development of metabolic syndrome and cardiovascular disease. Particularly, the fall of PRL levels below the lower range of normality during medical treatment with dopamine agonists appears to be associated with impaired gluco-insulinemic and lipid profile, and to promote weight gain, visceral obesity, insulin-resistance, diabetes mellitus, dyslipidemia, chronic inflammation, and sexual dysfunction, thus leading to the increase in overall cardiovascular risk. Based on this evidence, therapeutic choices should be shaped taking into due account this association in routine clinical practice. Special attention should be paid to patients with prolactinomas requiring chronic treatment with dopamine agonists, in whom over the course of the years the accomplishment of suppressed of strongly reduced PRL levels has been interpreted as protective against tumour recurrence, thus encouraging the long-term maintenance of dopamine agonist doses able to drive PRL levels below the normal range. In such patients, proper dopamine agonist dose adjustments should be promptly applied to restore normoprolactinemia in order to prevent or at least minimize metabolic imbalance and reduce the risk of cardiovascular diseases. In turn, patients at high cardiometabolic risk should be screened for potential hypoprolactinemia. However, the lack of an agreed biochemical definition of PRL deficiency may in fact limit the early identification of subjects at greater risk of cardiometabolic diseases.

Future research will better elucidate the role and the burden of hypoprolactinemia on cardiovascular morbidity and mortality, also providing an accurate biochemical definition for PRL deficiency, helpful to driving endocrinologists through the choice of the best individualized therapeutic approaches in clinical settings.

## Data Availability

No datasets were generated or analysed during the current study.
